# A novel NGS-based microsatellite instability (MSI) status classifier with 9 loci for colorectal cancer patients

**DOI:** 10.1186/s12967-020-02373-1

**Published:** 2020-05-28

**Authors:** Kai Zheng, Hua Wan, Jie Zhang, Guangyu Shan, Ningning Chai, Dongdong Li, Nan Fang, Lina Liu, Jingbo Zhang, Rong Du, Qixi Wu, Xichuan Li, Chunze Zhang

**Affiliations:** 1 Department of Colorectal Surgery, Tianjin Union Medical Center, Tianjin, 300121 China; 2 Beijing USCI Medical Laboratory, Beijing, 100195 China; 3 Department of Breast Oncology, Tianjin Medical University Cancer Institute and Hospital, National Clinical Research Center for Cancer, Tianjin’s Clinical Research Center for Cancer, Key Laboratory of Cancer Prevention and Therapy, Tianjin Medical University, Tianjin, 300060 China; 4 Tianjin Key Laboratory of Animal and Plant Resistance, College of Life Sciences, Tianjin Normal University, Tianjin, 300387 China; 5 State Key Laboratory of Medicinal Chemical Biology, Nankai University, Tianjin, 300070 China

**Keywords:** Microsatellite instability (MSI), NGS, Colorectal cancer (CRC), Immunotherapy, Mismatch repair, *TCF7L2*, *BRAF*, *KRAS*

## Abstract

**Background:**

With the recent emergence of immune checkpoint inhibitors, microsatellite instability (MSI) status has become an important biomarker for immune checkpoint blockade therapy. There are growing technical demands for the integration of different genomic alterations profiling including MSI analysis in a single assay for full use of the limited tissues.

**Methods:**

Tumor and paired control samples from 64 patients with primary colorectal cancer were enrolled in this study, including 14 MSI-high (MSI-H) cases and 50 microsatellite stable (MSS) cases determined by MSI-PCR. All the samples were sequenced by a customized NGS panel covering 2.2 MB. A training dataset of 28 samples was used for selection of microsatellite loci and a novel NGS-based MSI status classifier, USCI-msi, was developed. NGS-based MSI status, single nucleotide variant (SNV) and tumor mutation burden (TMB) were detected for all patients. Most of the patients were also independently detected by immunohistochemistry (IHC) staining.

**Results:**

A 9-loci model for detecting microsatellite instability was able to correctly predict MSI status with 100% sensitivity and specificity compared with MSI-PCR, and 84.3% overall concordance with IHC staining. Mutations in cancer driver genes (*APC*, *TP53*, and *KRAS*) were dispersed in MSI-H and MSS cases, while BRAF p.V600E and frameshifts in *TCF7L2* gene occurred only in MSI-H cases. Mismatch repair (MMR)-related genes are highly mutated in MSI-H samples.

**Conclusion:**

We established a new NGS-based MSI classifier, USCI-msi, with as few as 9 microsatellite loci for detecting MSI status in CRC cases. This approach possesses 100% sensitivity and specificity, and performed robustly in samples with low tumor purity.

## Background

Microsatellites, also called short tandem repeats (STRs), are short repeated nucleotide sequences with unit length between 1 and 6 base pairs (bps), which are widely presented in the genome of eukaryotes [1]. Microsatellite instability (MSI) represents the nucleotide insertions or deletions in the microsatellite loci. Alterations in microsatellite regions usually arise during DNA replication. The DNA mismatch repair (MMR) proteins, e.g. MLH1, MSH2, MSH6 and PMS2, are responsible for the repair of MSI. The germline or somatic inactivation of MMR genes could in turn result in MSI. MSI has been observed in a large number of cancer types, with the highest incidence in colon and endometrial cancers [2, 3]. Germline mutations in MMR proteins are associated with the pathogenesis of Lynch syndrome, which accounts for approximately 20% MMR-deficient (dMMR) colorectal cancer (CRC) [4]. For sporadic CRC, somatic mutations in microsatellite loci were found in 10% to 15% of cases [5].

MSI-H/dMMR cancers are expected to harbor a large number of mutations that might be recognized as neoantigens [6, 7] and could enable the patient to be sensitive to immune checkpoint blockade therapies. Thus, MSI status has become an important biomarker for immunotherapy, along with PD-L1 and tumor mutational burden (TMB) [8–11]. Clinical trials have shown that patients with MSI had improved responses to anti-PD-1/PD-L1 drugs, so accurate and efficient determination of MSI status could help to guide clinicians in choosing immune-oncology therapy.

Conventional MSI detection methods include MSI-PCR or indirectly by immunohistochemistry (IHC) staining of MMR protein expression. MSI-PCR is performed by amplifying five or more microsatellite loci in tumor and paired normal tissues, and determines MSI status by comparing the repeat number between the paired samples, classified into high (MSI-H), low (MSI-L), and stable (MSS) types. Low tumor purity and serious degradation of DNA may influence the MSI-PCR test. MMR IHC is a test of evaluating the expression levels of four clinically relevant MMR proteins (MLH1, MSH2, MSH6, and PMS2). dMMR is defined as any of these MMR proteins being totally absent in the nuclear staining of tumor tissue while present in adjacent benign tissue. If all four proteins are present in the tumor tissue, it is considered MMR proficient (pMMR). However, the MMR-related markers included in clinical IHC staining did not cover all MMR relevant genes, which may result in the relatively low correlation between IHC and MSI-PCR.

As Next-Generation Sequencing (NGS) has become a mainstream technology in oncology, NGS-based MSI detection methods are emerging. The selection of microsatellite loci may greatly influence the performance of the MSI detecting tools. According to previous studies, mononucleotide repeats are more sensitive than dinucleotide ones [12, 13]. Additionally, microsatellites with 10-20 bp repeat unit are too long to induce the slippage of DNA polymerase [14]. The bioinformatics tools evaluating MSI include tools directly assessing microsatellite loci in DNA, such as MANTIS [15], mSINGS [16], and MSIsensor [17], while others indirectly assess MSI status by analyzing single nucleotide variants and microindel (e.g., MSIseq and MSIpred) [18, 19]. Here we used MANTIS, which set the tumor and matched normal tissues data as two vectors. An L1 norm was defined to characterize the degree of stability of every site in the case, and the average of the L1 norm of all sites was used for evaluating the MSI status of the sample. In this study, we developed a novel MSI classifier named USCI-msi based on NGS data with 9 microsatellite loci. The classifier shows 100% sensitivity and specificity in CRC samples. We also analyzed genomic alterations in MSI-H cases and the correlation between MSI and TMB.

## Materials and methods

### Patient and sample preparation

Sixty-four colorectal tumor and matched normal samples were collected from August 2018 to August 2019 and analyzed following approved by the Institutional Review Board at Tianjin Union Medical Center. Written informed consent forms were obtained from each participant. All methods used in this study were performed in accordance with the relevant guidelines and regulations of the NCCN Clinical Practice Guidelines in Oncology: Colon Cancer (2019.V4).

Tumor samples were fresh or formalin-fixed and paraffin-embedded, while the paired control samples were either tumor-adjacent tissues or peripheral bloods. Genomic DNA of tissue and peripheral blood samples were isolated using QIAamp DNA FFPE Tissue Kit (Qiagen, German) and TIANamp Blood DNA Maxi Kit (TIANGEN, China) according to manufacturer’ s instructions, respectively.

### MSI-PCR testing

MSI-PCR testing was performed using the MSI detection kit (Microread Genetics, China) according to the manufacturer’s instructions. The length of PCR fragments were detected on the ABI 3730xl Genetic Analyzer (Applied Biosystems, USA), and analyzed with the GeneMapper software version 4.0 (Applied Biosystems, USA). Samples were considered as MSI-H when instability was observed in two or more of the six mononucleotide repeat loci (NR-21, BAT-26, NR-27, BAT-25, NR-24, and MONO-27), and as MSS when instability in less than two loci was observed.

### IHC staining

IHC staining was assayed with IHC kits (OriGene, USA) for MLH1, MSH2, MSH6 and PMS2 separately, according to the manufacturer’s instructions. dMMR was defined when any of these MMR proteins were totally absent in the tumor tissue while presented in adjacent benign tissue. Tumor tissues presenting all MMR proteins were defined as pMMR.

### Next-generation sequencing

A custom-designed 2.2 Mb panel, covering exons and partial introns of cancer driver genes, hereditary cancer related genes and therapy-related genes, was used in this study. 50-100 ng of sheared genomic DNAs were subjected to library construction with an MGIEasy universal DNA library kit (MGI, China), then followed by hybrid capture using an xGen Hybridization and Wash Kit (IDT, USA). Libraries’ quality and concentration were determined using a LabChip^®^ GX Touch™ nucleic acid analyzer (PerkinElmer, USA) and a Qubit fluorometer 3.0 (Life Technologies, USA), respectively. Tumor-matched normal samples were also sequenced as controls. The qualified libraries were sequenced with 2 × 100 bp paired-end reads on a MGISEQ-2000 (MGI, China) platform. The paired-end reads were aligned to human reference genome GRCh37/hg19 using BWA-MEM (v0.7.17) [20] and single nucleotide variants (SNVs) were determined by VarScan (v2.4.3) [21]. TMB was assessed as described by Chalmers and colleagues [14].

### Development of USCI-msi

A novel MSI status classifier, USCI-msi, was developed using a training dataset of 28 samples which included 7 MSI-H and 21 MSS samples determined by gold standard MSI-PCR. Microsatellite loci were first identified across the human reference genome (GRCh37/hg19) by RepeatFinder, and then limited to our panel region. The mononucleotide homopolymers, including the six mononucleotide loci used in the MSI-PCR test, were selected and analyzed in the training dataset with MANTIS using the default setting [15]. Low-quality paired-end reads were filtered out by length < 35 bp and base quality < 25. Low-quality microsatellite loci were filtered out by average base quality < 30 and minimum coverage < 30×, repeat type for a microsatellite locus < 3. The average instability scores for each locus in MSI-H and MSS samples were sorted in descending order. The loci that overlapped among the top 50 loci in the MSI-H cases and the bottom 50 loci in the MSS cases were chosen as marker microsatellite loci and reanalyzed in the training dataset with MANTIS. The NGS-based MSI detection method with the marker microsatellite loci was named USCI-msi classifier, and its performance was then validated with another 36 CRC samples.

### Statistical analysis

The difference between MSS and MSI-H cases were determined by Fisher’s exact test or non-parametric Mann–Whitney U test. Two-sided p < 0.05 was considered statistically significant.

## Results

### Evaluation of MSI status with USCI-msi

**Fig. 1:**
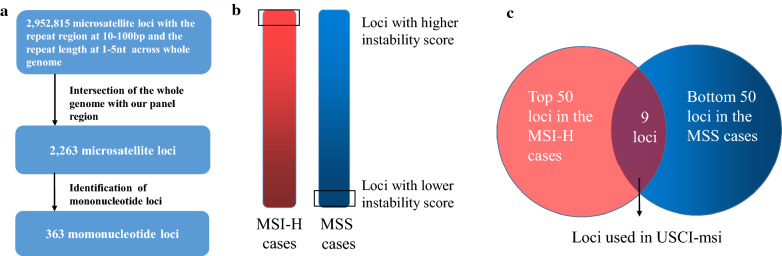
Schematic illustration of the selection pipeline (**a**–**c**) for microsatellite loci. 363 mononucleotide loci in our panel region were selected (**a**) and used for detecting MSI status. The 363 microsatellite loci were then sorted in descending order by the mean instability score calculated by MANTIS in MSI-H and MSS cases (**b**). The overlap of the top 50 loci in MSI-H cases and the bottom 50 loci in MSS cases was chosen for training USCI-msi (**c**)

There were 2,952,815 microsatellite loci identified over the genome with repeat region across 10 bp to 100 bp and repeat length across one to five (Fig. 1). 2,263 microsatellite loci were localized in the targeted region of our customized NGS panel. Since mononucleotide microsatellites were shown to be more sensitive in traditional MSI detection scenarios [12, 13], 363 mononucleotide repeat loci were selected for the downstream analysis.

**Fig. 2:**
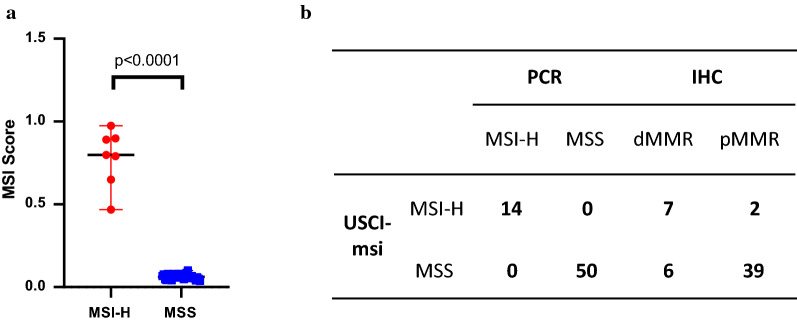
The performance of USCI-msi classifier. **a** USCI-msi was evaluated in the validation cohort (N = 36). MSI-H and MSS cases were grouped by MSI-PCR. The MSI status recognized by USCI-msi was consistent with those by MSI-PCR at the cutoff of 0.4. The mean MSI score of MSI-H samples (0.78, range 0.47–0.97) was significantly higher than that of MSS samples (0.06, range 0.04–0.10) (p < 0.0001). **b** A comparison among USCI-msi, MSI-PCR and IHC in the combination of training and validation cohorts. All MSI status recognized by USCI-msi were consistent with those by MSI-PCR, though only 85.2% (46/54) with IHC

We first evaluated the performance of the 363-loci classifier. Among the 28 samples in the training set, which included 7 MSI-H and 21 MSS cases, only one MSI-H sample was misjudged and defined as MSS. Then we analyzed the average instability scores of microsatellite loci in the MSI-H and MSS samples separately, and nine loci were the overlap between the top 50 loci in the MSI-H cases and the bottom 50 loci in the MSS, which may have the strongest discrimination power (Fig. 1). The training set samples were reanalyzed with the nine loci, which reached 100% accuracy, and then the nine loci MSI detection method was named USCI-msi classifier. The performance of USCI-msi was further evaluated in a CRC validation cohort (N = 36). The mean MSI score of MSI-H samples (0.78, range 0.47–0.97) was significantly higher than that of MSS samples (0.06, range 0.04–0.10) (Fig. 2a).

### A comparison among USCI-msi, MSI-PCR and IHC

All MSI status recognized by USCI-msi was consistent with those by MSI-PCR. The overall percent agreement (OPA) relative to MSI-PCR was 100% (64/64) in the combination of training and validation cohorts (Fig. 2b). As for MMR IHC staining, the results of MSI-NGS, MSI-PCR and IHC were not fully concordant (85.2%, 46/54) (Fig. 2b). Two pMMR samples were considered to be MSI-H by NGS and PCR methods. Then a closing inspection of the panel sequencing data of these two samples was made, and alterations of *POLE/POLD1* were found in both cases (Additional file 1: Table S2). Moreover, one also harbored alterations in three mismatch repair genes *MLH1*, *MSH6*, and *PMS2* (Additional file 1: Table S2). These indicated MMR deficiency may be caused by alterations in other related genes, or detrimental alterations which may lead to functional loss in MMR proteins, though normal expression may be retained. Six dMMR cases were evaluated as MSS by USCI-msi, though they were all MSH2-deficient. There was no alteration in *MLH1*, *MSH2*, *MSH6* and *PMS2* genes in these six cases, indicating deficiency of MSH2 may be caused by epigenetic inactivation of *MSH2* or other unknown reasons [22]. It may also be an early event, which had no effect on MSI. However, cases which were free of one or more of MLH1, MSH6 and PMS2 proteins were detected as MSI-H.

### The correlation of MSI status with patients’ clinical characteristics

The clinical characteristics of all the patients in this study are summarized in Table 1. The mean age of patients with clear information was 60.11 ± 11.67, ranging from 32-87. Two (2/57, 3.5%) patients were younger than 40 years, 27 (27/57, 47.37%) patients were between 40 and 60 years, and 28 (28/57, 49.12%) patients were older than 60 years. Patients with tumor stage I, II, III, and IV accounted for 9.43% (5/53), 39.62% (21/53), 49.06% (26/53) and 1.89% (1/53), respectively. The incidences of right hemicolon cancer, left hemicolon cancer and rectum cancer were 16% (8/50), 46% (23/50) and 38% (19/50), respectively. Clinical characteristics associated with MSI status were then examined: Patients aged between 40 and 60 years or with a tumor located at the right hemicolon were more likely to be MSI-H (p = 0.0174 and p = 0.0001, respectively). There was no statistically significant difference between the results for gender and tumor stage in MSI-H and MSS samples.

**Table 1:** Characteristics of patients in this study

	Total (N = 64)	MSI-H (N = 14)	MSS (N = 50)	*p* value
Age				0.0174
< 40	2	0	2	
40–60	27	11	16	
> 60	28	3	25	
Missing	7	0	7	
Gender				0.2241
Female	24	8	16	
Male	33	6	27	
Missing	7	0	7	
Clinical stage				0.2248
I	5	0	5	
II	21	6	15	
III	26	2	24	
IV	1	0	1	
Missing	11	6	5	
Cancer location				0.0005
Right hemicolon	8	*5*	3	
Left hemicolon	23	1	22	
Rectum	19	1	18	
Missing	14	7	7	

MSI-H and MSS were grouped by USCI-msi. Fisher’s exact test was used and patients with missing information were removed

*MSI-H* microsatellite instability-high, *MSS* microsatellite stability

### The performance of USCI-msi classifier on low tumor content samples

To estimate the performance of the USCI-msi classifier at low sample purity, two MSI-H samples with tumor contents of 32% and 67% were selected for gradient dilution experiments. As shown in Table 2, the MSI score correlated with the tumor content along with the dilution with the matched normal tissue DNA. When diluted to 50%, all mixtures were classified as MSI-H, and the sample with the higher score was still confirmed as MSI-H at 33% dilution. Based on the dilution factor, the MSI classifier is robust to the tumor content as low as 16%.

**Table 2:** Dilution assay

Dilution, %	Tumor purity, %	MSI score	MSI status
100	32	0.87	MSI-H
50	16	0.46	MSI-H
33	10.6	0.26	MSS
20	6.4	0.20	MSS
100	67	1.26	MSI-H
50	33.5	0.67	MSI-H
33	22.3	0.42	MSI-H
20	13.4	0.28	MSS

Two MSI-H samples with tumor contents of 32% and 67% were selected for gradient dilution experiments by diluting tumor DNAs with their matched normal DNAs. The mixtures were tested for MSI status by USCI-msi

*MSI-H* microsatellite instability-high, *MSS* microsatellite stability

### Genomic alterations across MSI-H tumors

**Fig. 3:**
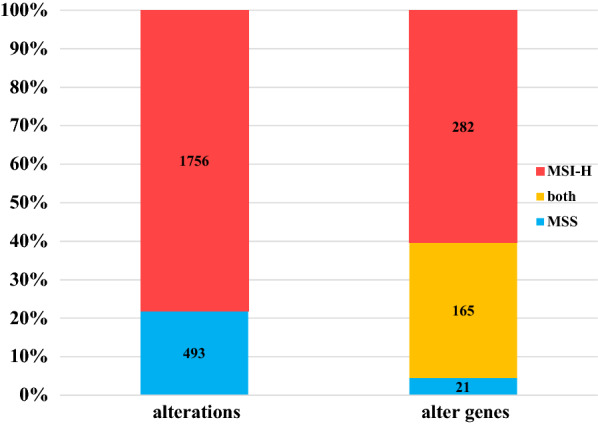
Schematic illustration of altered sites and genes in 64 colorectal cancer cases

**Fig. 4:**
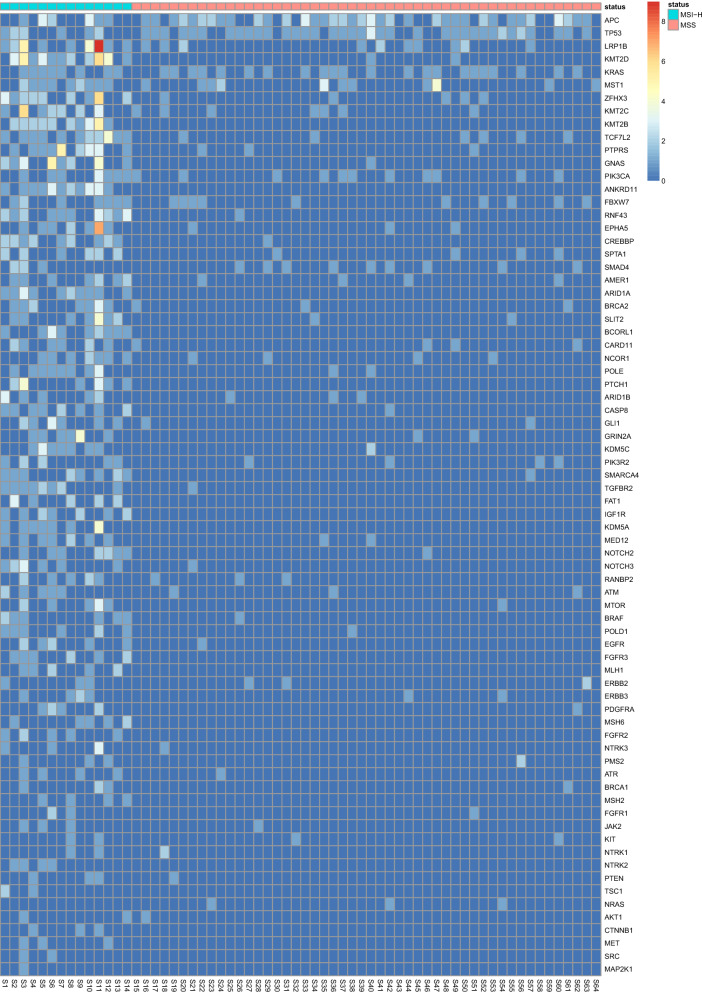
Hotspot mutant genes in 64 colorectal cancer cases. The most frequent mutant genes are listed in descending order. Colour bar, mutant frequency of genes in a sample

There were total 2249 alterations across 468 genes in 64 CRC cases. Alterations were significantly enriched in MSI-H samples, with 78% (1756 alterations in 447 genes) found in 14 MSI-H samples, while 22% (493 alterations in 186 genes) were found in 50 MSS samples (Fig. 3). The mean number of alterations was 125 (range 63-302) for MSI-H cases, and 10 (range 1-26) for MSS. 60.2% (282/468) of the genes were only affected in MSI-H cases, while only 4.5% (21/468) in MSS (Fig. 3). At gene level, the top mutated genes only in MSI-H cases included *ANKRD11* (78.6%, 11/14), *ARID1A* (71.4%, 10/14), *KMT2B* (71.4%, 10/14), *BCORL1* (64.3%, 9/14), *IGF1R* (50%, 7/14), *KDM5* (50%, 7/14), *POLD1* (50%, 7/14) and *TSC1* (50%, 7/14) (Additional file 1: Table S2 and Fig. 4). Alterations in the hot genes of CRC, *APC*, *TP53* and *KRAS*, were common in both MSI-H and MSS cases (Additional file 1: Table S2 and Fig. 4).

There were 90 recurrent mutations in 85 genes, of which the most frequent ones were p.E384fs in *TCF7L2* (NM_001198530), p.G659fs in *RNF43* (NM_017763) and p.E125fs in *TGFBR2* (NM_003242) (Table 3). All of these three mutations were located at mononucleotide repeat regions and were detected in 10, 7 and 6 MSI-H cases, respectively, which indicated that microsatellite loci were commonly unstable in MSI-H cases [23–25]. Hot mutations, p.G12V/S/D/A, p.G13D and p.A146T of KRAS, were found in both MSI-H and MSS cases, while BRAF p.V600E were only found in 4 MSI-H cases (Table 3), which indicated that BRAF p.V600E may be correlated with MSI [26].

**Table 3:** Hotspot mutations in colorectal cancer cases

Gene	Hotspot	Number of variant cases		
		Total	MSI-H	MSS
KRAS	p.G12V/S/D/A	16	3	13
	p.G13D	7	5	2
	p.Q61H	2	0	2
	p.A146T	4	3	1
NRAS	p.Q61R/H/L	3	0	3
BRAF	p.V600E	4	4	0
TCF7L2	p.E384fs	10	10	0
RNF43	p.G659fs	7	7	0
TGFBR2	p.E125fs	6	6	0
QKI	p.K132fs	4	4	0
CARD11	p.R555fs	3	3	0
BCORL1	p.S1679fs	3	3	0
JAK3	p.Q39fs	2	2	0
BCOR	p.Q1156fs	2	2	0

p.G12V/S/D/A, p.G13D and p.A146T of KRAS were found in both MSI-H and MSS cases, while BRAF p.V600E was only found in 4 MSI-H cases. p.E384fs of TCF7L2 were highly mutated in MSI-H samples

*fs* fragment shift

Eleven of MSI-H (78.6%, 11/14) and one of MSS (2%, 1/50) cases carried mutations in the four MMR genes, *MLH1*, *MSH6*, *MSH2* and *PMS2* (Fig. 4). Mutations in these four genes were found in five, five, four and four MSI-H samples, respectively. Four cases were detected with mutations in two or more of these genes. All of above indicated that MMR-related genes were highly mutated in MSI-H samples.

**Fig. 5:**
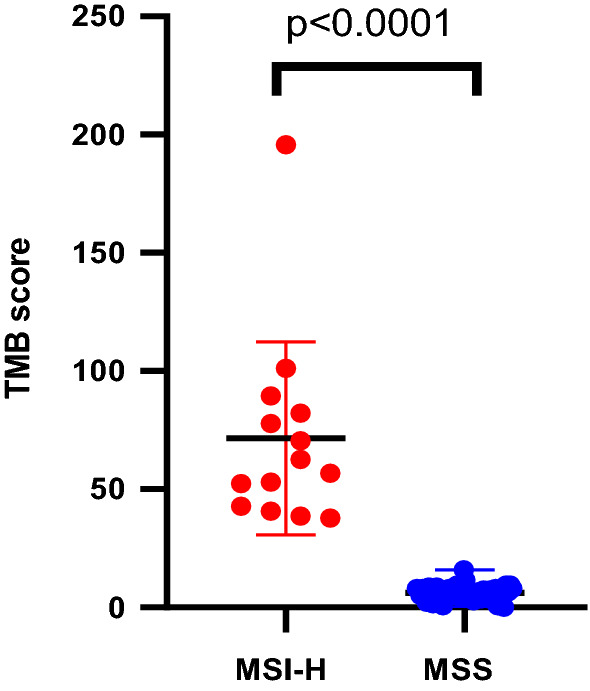
MSI-H correlated with high TMB in colorectal cancer patients. MSI-H tumors had a significantly increased mean TMB (59.65 mutations/Mb) compared to MSS samples (6.15 mutations/Mb) (p < 0.0001)

In all the CRC samples, MSI-H tumors had a significantly increased mean TMB (59.65 mutations/Mb) compared to MSS samples (6.15 mutations/Mb) (Fig. 5). The minimal TMB score of MSI-H samples (37.8 mutations/Mb) far outstripped the top TMB score of MSS samples (16 mutations/Mb). Thus, MSI status was also highly correlated with TMB (p < 0.0001, Fig. 5).

## Discussion

With the increasingly routine adoption of clinical NGS panels to oncology, it is crucial to profile different genomic variations by the integration of multiple components in a single assay, which could make full use of the limited tissues and simplify procedures. The panel used in this study covered more than 600 genes, including most cancer-related genes, which was sufficient for genomic profiling across diverse tumor types, including CRC. In this study, we present a novel NGS-based tool, USCI-msi, to detect MSI status. This method achieved 100% sensitivity and 100% specificity in CRC samples, which is comparable to or better than the recent reports [27–30]. Pang et al. developed a decision tree classifier model and was able to correctly predict the MSI status of 112 clinical cases with 100% sensitivity and specificity using 8682 mononucleotide and dinucleotide repeat loci [27]. A PCA method was used to generate an MSI score for stratification of MSI-H and MSS patients from a NGS comprehensive genomic profiling assay (FoundationOne and FoundationOneHeme panel). The sensitivity of this method was 97.0% when compared to corresponding MSI-PCR and IHC [28]. In a cohort with 2189 matched cases, the MSI-NGS method with 7317 target microsatellite loci had a sensitivity of 95.8%, specificity of 99.4%, positive predictive value of 94.5%, and negative predictive value of 99.2% as compared to MSI-PCR [29]. These methods based on target capture sequencing included most of the microsatellite loci in the target region. Gallon et al. presented a single molecule molecular inversion probe and sequencing-based MSI assay with six loci and achieved 100% concordance with the MSI-PCR in 220 CRCs [30]. However microsatellite loci selection of this modified amplicon sequencing method was limited to a small amount of candidate loci. USCI-msi with nine microsatellite loci, accompanied genomic profiling assay, showed a better performance than the unselected 363-loci set. Some of the genes covering the 9 loci have been previously reported frequently mutated in MSI-H tumors (e.g., microsatellites in *POLD1*, *EP300*) [27, 31]. The six mononucleotide repeat sequences used in MSI-PCR were also included in the 363-loci classifier, but were absent in the final classifier. Our data also proved that fewer than 10 loci were sufficient to classify MSI status in CRC cases, but the selection of the loci should adjust to the NGS panel used.

We also analyzed alterations in our Chinese cohort. There were many more alterations in MSI-H cases than in MSS cases, though cancer driver genes such as *APC*, *TP53*, and *KRAS* are commonly mutated in CRC samples, regardless the MSI status. The most frequent mutation in MSI-H cases was *TCF7L2* p.E384fs. Frameshift mutations in *TCF7L2* gene had been found in colorectal and gastric carcinomas with high MSI [23, 24]. TCF7L2 is an important member in the Wnt signaling pathway and mutations in Wnt-related genes were also found to be enriched in MSI-H cases in a cohort of 67,000 pan-tumor cases [28]. The mismatch repair genes were highly mutated in MSI-H samples, which indicated MSI was a result of MMR gene dysfunction. However, the relatively low concordance between USCI-msi and IHC confirmed that loss of MSH2 protein didn’t always result in MSI.

MSI has become a promising biomarker for predicting therapeutic response to immune checkpoint inhibitors. Recently, pembrolizumab was approved for all types of solid tumors exhibiting MSI-H. Consistent with previous studies, MSI-H cases had higher TMBs than the MSS cases in our study. Metastatic colorectal cancer with high MSI has a good response to immunological checkpoint inhibitor therapies [8–11]. Tumors with high MSI may contain abundant new antigens that can elicit an immune response; thus, determining MSI status offers an opportunity to identify patients who may benefit from immunotherapy. In this study, USCI-msi classifier was only tested in CRC cases. In the future, it will be evaluated on more cancer types.

## Conclusion

We described a new NGS-based MSI classifier, USCI-msi, with as few as 9 microsatellite loci for detecting MSI status in CRC cases. This approach possesses 100% sensitivity and specificity, and performed robustly in samples with low tumor purity.

## Supplementary information

**Additional file 1: Table S1.** Alterations in two pMMR samples which were considered MSI-H by USCI-msi. **Table S2.** Gene enrichment in MSI-H or MSS cases. Genes with ten or more alterations in the colorectal cancer cases are listed. Genes with alterations only in MSI-H cases are in bold.

## Data Availability

The datasets used during the current study are available from the corresponding author on reasonable request.

## Abbreviations

CRC: Colorectal cancer
MSI: Microsatellite instability
MSI-H: MSI-high
MSI-L: MSI-low
MSS: Microsatellite stability/stable
IHC: Immunohistochemistry
pMMR: Mismatch repair (MMR) proficient
dMMR: MMR-deficient
STRs: Short tandem repeats
SNV: Single nucleotide variant
TMB: Tumor mutational burden
